# Cracking the code: uncovering the factors that drive COVID-19 standard operating procedures compliance among school management in Malaysia

**DOI:** 10.1038/s41598-023-49968-4

**Published:** 2024-01-04

**Authors:** Noor Sakinah Ahmad, Karmegam Karuppiah, Sarva Mangala Praveena, Nina Fatma Ali, Murugadas Ramdas, Nur Athirah Diyana Mohammad Yusof

**Affiliations:** 1 Department of Environmental and Occupational Health, Faculty of Medicine and Health Sciences, Universiti Putra Malaysia, Serdang, Malaysia; 2 Politeknik Sultan Salahuddin Abdul Aziz Shah, Shah Alam, Malaysia; 3 Engineering and Technology Department, Razak Faculty of Technology and Informatics, Universiti Teknologi Malaysia, Kuala Lumpur, Malaysia

**Keywords:** Infectious diseases, Risk factors

## Abstract

Malaysia's government's decision to reopen schools during the COVID-19 outbreak, especially for students taking important exams, has alarmed the public. However, the Ministry of Education has implemented a COVID-19 Standard Operating Procedure (SOP) for educational institutions. The school management’s ability to protect children from COVID-19 rests on their understanding, attitudes, and practices regarding COVID-19 SOP compliance. This study investigated Selangor, Kuala Lumpur, and Putrajaya school management’s COVID-19 SOP compliance determinants. Multistage sampling was used to sample 740 school management from Kuala Lumpur, Putrajaya, and Selangor. A self-administered questionnaire collected sociodemographic, occupational, and lifestyle data, knowledge, attitude, and practice of COVID-19 SOP compliance. The school management had good knowledge, attitude, and practice toward COVID-19 SOP. Monthly income, school location, smoking status, and physical activity differed significantly from KAP (*p* < 0.05). The correlation between KAP showed a significant relationship with the values (r = 0.348, *p* < 0.001) and (r = 0.358, *p* = 0.003). Nine independent variables strongly predicted SOPs compliance practice in multiple linear regression: knowledge, attitude, age, source of knowledge; Ministry of Health, physical activities, type of infectious disease exposed; Tuberculosis and Measles (*p* < 0.05). The data indicate that school management exhibits good knowledge, attitude, and compliance with SOPs during the pandemic. School management oversees SOPs, and to keep schools safe, management must analyse hazards and take action. Therefore, knowledge and attitude are expected to determine factors of practice toward COVID-19 SOP compliance.

## Introduction

COVID-19 has rapidly become a threat to global public health since the outbreak in Wuhan, China, in December 2019 and has contributed to massive worldwide socio-economic disruption. COVID-19 is highly contagious; apart from that, the main symptoms are fever, dry cough, fatigue, myalgia, and dyspnea^1^. Thus, this disease recently emerged as a significant global epidemic and has caused an important public health issue. As a result, COVID-19 was declared a public health emergency of international concern (PHEIC) on January 30, 2020, and the WHO subsequently declared a global pandemic on March 12, 2020^2^. The government declared a Movement Control Order (MCO) on March 16, 2020. Subsequently, the MCO period has been extended to several phases, including the Conditional Movement Control Order (CMCO) and the Rehabilitation Movement Control Order (RMCO). The second wave of COVID-19 arose after the Sabah state election on September 26, 2020^3^. Meanwhile, on January 13, 2021, the Prime Minister of Malaysia announced MCO 2.0 for the whole state to curb the spread of COVID-19^4^. After the sudden increasing number of COVID-19 cases, which rose to 9,020 per day on May 29, 2021, the Prime Minister announced a total lockdown to all states in Malaysia from June 1, 2021, until June 14, 2021^5^.

Children and teenagers below 18 are prone to be infected and have a severe reaction to COVID-19 likewise, the school staff with underlying medical conditions and above 60 years old. School management is responsible for managing SOP in the school area. School management must be able to assess the risks and execute appropriate actions to maintain a conducive and safe environment. According to the Director General of Health, Dr. Noor Hisham stated that between January 1 and May 7, 2021, a total of 138 clusters involving 9,330 cases were identified. These clusters included educational institutions under the Ministry of Education (MOE), private institutions registered with the MOE, and higher education institutions. From overall clusters, MOE educational institutions had the most, with 72 clusters including 3,520 cases, followed by other educational institutions with 41 clusters (3,647 cases)^6^. Poor SOP management leads to rising COVID-19 cases in schools. With the number of COVID-19 cases in schools increasing, it is thought that not practicing primary infection control is the main reason infectious diseases spread^7^. In the meantime, knowledge, and attitude significantly affect how COVID-19 is prevented in practice.

Despite being conducted during the later stages of the COVID-19 pandemic, this study holds significant value as it offers valuable insights and resourceful input for the ongoing enhancement of the existing guidelines. Even though COVID-19 has entered an endemic phase, it still poses health hazards, and following SOPs is important for reducing the virus's spread. The school management has a responsibility to ensure a safe learning environment for the students and school staff.

In order to maintain effective preventative measures and reduce the possibility of outbreaks inside schools, it is important to understand the variables that affect their compliance. Guidelines and methods for COVID-19 may change in the future due to the development of new variations and expanding scientific understanding. It gives information on their capacity to keep current and make the required changes to maintain a secure learning environment. Examining school management's compliance throughout the endemic era offers important information regarding their readiness and capability to address upcoming health emergencies or disease outbreaks. The results can help policymakers and educational authorities improve school health policies, practices, and equality while also ensuring that schools are well prepared to handle any foreseeable public health emergency.

In a study conducted by Abu Hasan et al.^8^ among Malaysian residents there is still a need to improve KAP among Malaysians. Nevertheless, it was found that knowledge score is high at 91.4%, but the behaviour and practice are low. On the part of promoting public awareness and understanding of COVID-19, clear communication from the authorities is nevertheless essential^9^. Therefore, it is necessary to investigate the association between sociodemographic traits, lifestyle, and occupational factors and the KAP. This study intends to examine the knowledge, attitude, and compliance with COVID-19 SOPs among school management and the factors linked with their compliance practices. Thus, knowledge and attitude are expected to determine factors of practice toward COVID-19 SOP compliance.

## Data and methods

### Study design and sampling

**Figure 1:**
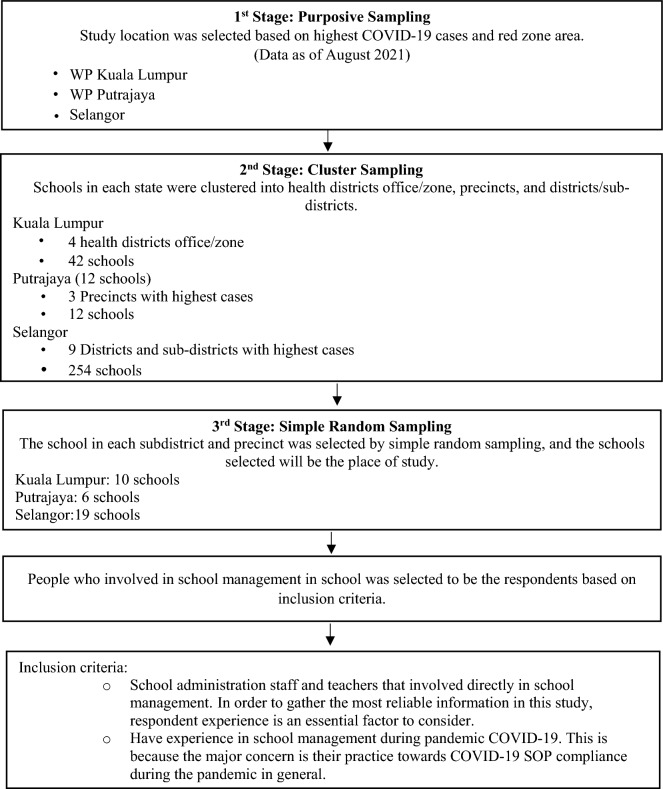
Multistage sampling method process.

A cross-sectional study was carried out among school administrators and teachers in Kuala Lumpur, Putrajaya, and Selangor government primary and secondary schools. This study sampling was conducted during the later stage of the pandemic from December 2021 until March 2022. The sample was selected using a multistage sampling method in which the schools were clustered into health districts zone, precincts and districts/subdistricts based on the highest COVID-19 cases (data as of August 2021), then randomly sampled using random number generators; a total of 35 schools from three states were chosen (refer to Fig. 1). After that, 740 respondents were picked from the sampled schools. The response rate was 92.5%. A systematic way for collecting data from a large, diverse population is multistage sampling. This sampling method is also used due to constraints in terms of time and resources.

### Study instruments and procedures

A set of self-administered questionnaires via a Google form, which was adapted from previous studies as the research instruments to achieve the objectives, was used in this study^8–13^. After getting approval from the school principals, the Google form link was shared with the school administration via WhatsApp and Telegram. The information was collected after respondents submitted the Google form.

These self-reported questionnaires were used to determine the determinant factors of practicing COVID-19 SOP compliance among school management in Selangor, Kuala Lumpur, and Putrajaya. The questionnaire comprised six components: Section A: Sociodemographic backgrounds, Section B: lifestyle, Section C: occupational factors, Section D: Knowledge, Section E: Attitude, and Section F: Practice. The KAP questionnaire includes questions on knowledge about COVID-19, its transmission, symptoms, and SOP, Attitude towards COVID-19, and practices towards COVID-19. All knowledge and attitude factors were measured using a 5-point Likert scale (5 = Strongly Agree, 4 = Agree, 3 = Neither agree nor disagree, 2 = Disagree, and 1 = Strongly Agree) while practice was measured using (5 = Always, 4 = Often, 3 = Sometimes, 2 = Rarely, and 1 = Never). The total mean scores for KAP level were determined by dividing the mean scores with number of scores^14^. The mean scores defined the KAP level (low = 1–2.33, medium = 2.34–3.67, and high = 3.68–5). The scoring rate was determined by dividing the actual mean score by the overall mean score and multiplying that result by 100^15^. Besides, information regarding respondent demographic details was also asked. A pre-test was conducted among 30 school administrators and teachers from non-sampled schools and the value obtained for Cronbach’s alpha was 0.826 which showed that this questionnaire was reliable.

The content validation was sent to four experts and the experts specialised in Health Risk Management, Public Participation, Occupational, and Environmental Health. For content validity, the number of experts should not be fewer than 2, but not more than 6^16,17^. For S-CVI Results (relevancy of the comprehensive questionnaire), the S-CVI/UA = 0.82 and the S-CVI/AV = 0.91. The Universal Agreement (UA) is calculated by adding all I-CVI’s equal to 1.00 (75 items) divided by 91, while the Average (AV) takes the sum of all I-CVI (83.25) divided by 91. Overall, the UA method and the AV approach show high content validity of the questionnaires. With S-CVI/AV = 0.90, the instrument had good face validity and good content-related validity^18^.

### Data analysis

The collected data from the questionnaire and measurement were analysed using IBM SPSS software (Version 25) to determine the determinant factors of practicing COVID-19 SOP compliance among school managements in Selangor, Kuala Lumpur, and Putrajaya. The tests that were used in this study were descriptive analysis, independent t-test, ANOVA, Pearson correlation, and Multiple Linear Regression.

### Human subject approval statement

Ethics approval was obtained from the Ethics Committee of Research Involving Human Subjects, Universiti Putra Malaysia (JKEUPM-2022-036). Moreover, permission to conduct the study in the selected schools had been obtained from the Ministry of Education (MOE) (KPM.600-3/2/3-eras (11496)), the State Education Department (JPWPKL.600-9/1/5Jld.4(64)) (JPWPP.100-2/2/2Jld.9(71)) (JPNS.SPD.600-1/1/2JLD.15(68)), and the school principals. All procedures related to human participants were performed following the ethical standards of the Helsinki Declaration. Each respondent was given an informed consent form together with the questionnaires to read and sign before filling up the questionnaires form via Google form. The informed consent form states that the respondent's participation in this study is voluntary without force from other parties. It also stated in the form the purpose of the study, the steps they need to undergo in this study, the benefits, and possible risks.

## Results

### Sociodemographic characteristics, lifestyle, physical activity, and occupational factors of the respondents

The respondents in this study were voluntary based; 740 were included. Table 1 shows the sociodemographic background of the respondents for the gender, age, race, monthly income, school location, and source of COVID-19 knowledge. Most of the respondents were female (72.3%), aged between 31 and 50 years old (65.4%), Malay (88.2%), and earned between RM4001 and RM6000 (47.8%). The majority of them work at school in Selangor (67.6%), and only a few come from Kuala Lumpur (21.9%) and Putrajaya (10.5%). The respondents mainly received information on COVID-19 from the Ministry of Health (88.0%), followed by social media (79.9%), Ministry of Education (76.9%), Friends (58.2%), Family (55.0%) and Ministry of Communication and Multimedia (52.8%).

**Table 1:** Differences between sociodemographic characteristics with knowledge, attitude, and practice (N = 740).

Variables	N	%	Knowledge		Attitude		Practice	
			Mean ± SD	*p*-value	Mean ± SD	*p*-value	Mean ± SD	*p*-value
Gender								
Male	205	27.7	4.61 ± 0.326	0.586	4.71 ± 0.514	0.023*	4.14 ± 0.450	0.045*
Female	535	72.3	4.57 ± 0.295		4.73 ± 0.354		4.12 ± 0.411	
Age								
20–30	96	13.0	4.54 ± 0.263	0.092	4.64 ± 0.606	0.064	4.12 ± 0.535	0.326
31–40	244	33.0	4.62 ± 0.299		4.76 ± 0.340		4.10 ± 0.411	
41–50	240	32.4	4.58 ± 0.327		4.73 ± 0.385		4.13 ± 0.368	
51–60	160	21.6	4.55 ± 0.298		4.71 ± 0.365		4.18 ± 0.438	
Race								
Malay	653	88.2	4.58 ± 0.294	0.042*	4.72 ± 0.396	0.042*	4.12 ± 0.423	0.428
Chinese	43	5.8	4.51 ± 0.488		4.60 ± 0.577		4.13 ± 0.475	
Indian	38	5.1	4.70 ± 0.162		4.83 ± 0.275		4.22 ± 0.365	
Others	6	0.8	4.58 ± 0.199		4.933 ± 0.275		4.28 ± 0.277	
Monthly income								
< RM4000	172	23.2	4.50 ± 0.333	0.0001*	4.61 ± 0.542	0.0001*	4.04 ± 0.515	0.010*
RM4001–RM6000	351	47.4	4.63 ± 0.288		4.80 ± 0.306		4.15 ± 0.374	
RM6001–RM8000	181	24.5	4.57 ± 0.301		4.71 ± 0.381		4.17 ± 0.384	
> RM8001	36	4.8	4.55 ± 0.247		4.63 ± 0.449		4.10 ± 0.500	
Chen et al. (2021)^38^; Mat Din et al., 2020;^39^								
Kuala Lumpur	162	21.9	4.53 ± 0.316	0.001*	4.68 ± 0.387	0.0001*	4.02 ± 0.435	0.001*
Putrajaya	78	10.5	4.50 ± 0.367		4.56 ± 0.681		4.18 ± 0.438	
Selangor	500	67.6	4.61 ± 0.286		4.76 ± 0.340		4.16 ± 0.410	
Source of COVID-19 knowledge								
Ministry of health								
No	89	12.0	4.65 ± 0.372	0.286	4.74 ± 0.441	0.490	4.17 ± 0.365	0.237
Yes	651	88.0	4.57 ± 0.292		4.72 ± 0.399		4.12 ± 0.429	
Ministry of education								
No	171	23.1	4.58 ± 0.323	0.172	4.63 ± 0.516	0.0001*	4.02 ± 0.496	0.002*
Yes	569	76.9	4.58 ± 0.299		4.75 ± 0.359		4.16 ± 0.392	
Ministry of communication and multimedia			4.58 ± 0.332	0.080	4.72 ± 0.411	0.556	4.10 ± 0.432	0.460
No	349	47.2	4.58 ± 0.278		4.72 ± 0.399		4.16 ± 0.412	
Yes	391	52.8						
Social media								
No	149	20.1	4.66 ± 0.308	0.629	4.76 ± 0.363	0.263	4.09 ± 0.455	0.091
Yes	591	79.9	4.56 ± 0.301		4.72 ± 0.413		4.14 ± 0.413	
Family								
No	333	45.0	4.61 ± 0.308	0.688	4.73 ± 0.380	0.562	4.10 ± 0.426	0.348
Yes	407	55.0	4.56 ± 0.300		4.72 ± 0.423		4.15 ± 0.418	
Friends								
No	305	41.2	4.60 ± 0.318	0.333	472 ± 0.412	0.530	4.11 ± 0.430	0.230
Yes	435	58.2	4.18 ± 0.294		4.73 ± 0.399		4.14 ± 0.416	

Significance at *p < 0.05.

For occupational factors, Table 2 shows that from a total of 740 respondents, 19.7% are school administrators, and the remaining 80.3% are teachers. Most respondents have worked for more than ten years (56.6%), have zero experience handling infectious diseases (71.6%), and manage infectious disease programs (73.2%) at schools. However, almost 62.0% of respondents have been exposed to infectious diseases at school. The result showed that measles (35.5%) has the highest number of reported diseases occurring at school, followed by dengue (27.3%), food poisoning (24.1%), Tuberculosis (7.4%), and HFMD (6.2%).

**Table 2:** Differences between occupational characteristics with knowledge, attitude, and practice (N = 740).

Characteristics	N	%	Knowledge		Attitude		Practice	
			Mean ± SD	*P*-value	Mean ± SD	*P*-value	Mean ± SD	*P*-value
Position								
School administrators	146	19.7	4.57 ± 0.297	0.557	4.72 ± 0.355	0.932	4.19 ± 0.373	0.060
Teacher	594	80.3	4.58 ± 0.306		4.73 ± 0.415		4.11 ± 0.433	
Year of services								
1–5 years	242	32.7	4.54 ± 0.263	0.092	4.64 ± 0.606	0.064	4.12 ± 0.535	0.326
6–10 years	79	10.7	4.62 ± 0.299		4.76 ± 0.340		4.10 ± 0.411	
> 10 years	419	56.6	4.58 ± 0.327		4.73 ± 0.385		4.13 ± 0.368	
Experience in handling infectious disease cases in school								
No	530	71.6	4.55 ± 0.308	0.0001*	4.71 ± 0.427	0.117	4.11 ± 0.425	0.023*
Yes	210	28.4	4.65 ± 0.283		4.76 ± 0.337		4.18 ± 0.384	
Experience in managing infectious disease program								
No	542	73.2	4.55 ± 0.309	0.0001*	4.71 ± 0.424	0.075	4.08 ± 0.438	0.0001*
Yes	198	26.8	4.65 ± 0.280		4.77 ± 0.339		4.27 ± 0.338	
Exposure to infectious diseases at school								
No	281	38.0	4.54 ± 0.292	0.002*	4.72 ± 0.400	0.797	4.11 ± 0.450	0.361
Yes	459	62.0	4.61 ± 0.309		4.73 ± 0.407		4.14 ± 0.404	
Type of infectious disease								
Dengue			4.56 ± 0.303	0.002*	471 ± 0.421	0.168	4.11 ± 0.445	0.059
No	538	72.7	4.64 ± 0.303		4.75 ± 0.354		4.62 ± 0.351	
Yes	202	27.3						
Tuberculosis (TB)								
No	685	92.6	4.57 ± 0.302	0.032*	4.72 ± 0.404	0.932	4.13 ± 0.426	0.409
Yes	55	7.4	4.66 ± 0.321		4.72 ± 0.405		4.08 ± 0.369	
HFMD								
No	694	93.8	4.58 ± 0.305	0.954	4.73 ± 0.404	0.197	4.13 ± 0.427	0.309
Yes	46	6.2	4.58 ± 0.290		4.65 ± 0.392		4.07 ± 0.346	
Food poisoning								
No	562	75.9	4.56 ± 0.294	0.002*	4.71 ± 0.407	0.164	4.11 ± 0.432	0.016*
Yes	178	24.1	4.64 ± 0.328		4.77 ± 0.394		4.20 ± 0.382	
Measles								
No	477	64.5	4.55 ± 0.316	0.002*	4.72 ± 0.396	0.929	4.14 ± 0.435	0.353
Yes	263	35.5	4.63 ± 0.276		4.73 ± 0.419		4.11 ± 0.397	

Significance at *p < 0.05.

Based on Table 3, the lifestyle factors included in this study were smoking status, physical activity status, if they experience “negative stress” or stress in daily life, and sleep disturbances. The majority of the respondents have never smoked before (90.3%), “sometimes” do physical activity (47.0%), and “rarely’ experienced stress in daily life (45.5%). Most of the respondents never have sleep disturbances in their daily life (41.6%).

**Table 3:** Differences between lifestyle characteristics with knowledge, attitude, and practice (N = 740).

Characteristics	N	%	Knowledge	Attitude	Practice
			Mean ± SD	Mean ± SD	Mean ± SD
Smoking status					
Never	668	90.3	4.53 ± 0.338	4.44 ± 0.806	4.06 ± 0.542
Rarely	29	3.9	4.66 ± 0.243	4.79 ± 0.309	4.11 ± 0.366
Sometimes	22	3.0	4.55 ± 0.330	4.71 ± 0.383	4.09 ± 0.411
Often	20	2.7	4.57 ± 0.294	4.74 ± 0.387	4.24 ± 0.381
Always	1	0.1	4.65 ± 0.203	4.77 ± 0.282	4.09 ± 0.670
Physical activities					
Never	36	4.9	4.53 ± 0.338	4.44 ± 0.806	4.14 ± 0.450
Rarely	136	18.4	4.66 ± 0.243	4.79 ± 0.309	4.12 ± 0.411
Sometimes	348	47.0	4.55 ± 0.330	4.71 ± 0.383	4.61 ± 0.326
Often	186	25.1	4.57 ± 0.294	4.74 ± 0.387	4.57 ± 0.295
Always	34	4.6	4.65 ± 0.203	4.77 ± 0.282	4.57 ± 0.295
Stress experience					
Never	129	17.4	4.54 ± 0.284	4.75 ± 0.361	4.15 ± 0.530
Rarely	337	45.5	4.57 ± 0.305	4.74 ± 0.349	4.12 ± 0.408
Sometimes	236	31.9	4.61 ± 0.315	4.69 ± 0.488	4.11 ± 0.3386
Often	36	4.9	4.63 ± 0.285	4.66 ± 0.427	4.19 ± 0.2330
Always	2	0.3	5.00 ± 0.001	5.00 ± 0.001	4.67 ± 0.001
Sleep disturbances					
Never	308	41.6	4.56 ± 0.298	4.71 ± 0.438	4.17 ± 0.476
Rarely	289	39.1	4.62 ± 0.2254	4.76 ± 0.352	4.11 ± 0.392
Sometimes	118	15.9	4.52 ± 0.406	4.64 ± 0.438	4.07 ± 0.326
Often	23	3.1	4.57 ± 0.300	4.70 ± 0.334	4.07 ± 0.424
Always	2	0.3	5.00 ± 0.001	5.00 ± 0.001	4.67 ± 0.001
Scoring Rate (%) for lifestyle characteristics			91.8 4.59 ± 0.059	93.8 4.76 ± 0.140	82.4 4.22 ± 0.256

### Knowledge of COVID-19 SOP compliance

The results revealed that respondents had a high knowledge of COVID-19 SOPs compliance, with an overall mean of 4.58 ± 0.304 and a scoring rate of 91.6% (Supplementary Table 1). Most of the respondents have a high level of knowledge about COVID-19 (4.88 ± 0.013), COVID-19 transmission (4.82 ± 0.050), COVID-19 symptoms (4.76 ± 0.152), and COVID-19 standard operating procedure (4.28 ± 1.038). However, eating at the canteen during the break and COVID-19-related absenteeism as a non-disciplinary issue scored lowest at 42.2% (2.11 ± 1.381) and 46.1% (2.30 ± 1.442), respectively (Supplementary Table 3). Based on Table 1, knowledge has significant mean differences across races, monthly income, and school location. The level of knowledge also shows a significant difference in those who experience handling cases, managing programs related to infectious diseases, and being exposed to diseases like dengue, Tuberculosis, food poisoning, and measles (Table 2). For lifestyle characteristics of school management, knowledge showed high scoring rate compared to practice but slightly lower than attitude (Table 3).

### Attitude towards COVID-19 SOP compliance

The respondent’s attitude toward COVID-19 SOP compliance was considered high, with an overall mean of 4.72 ± 0.404 and a scoring rate of 94.4% (Supplementary Table 1). The highest scoring rate was on the belief of everyone’s responsibility to prevent the spread of COVID-19 (4.90 ± 0.409), which was 98.0%. Contradict that the respondents’ belief in the ability of vaccines to protect them from COVID-19 infection (4.55 ± 0.744) had the lowest scoring rate of 90.9% (Supplementary Table 4). Based on Table 1, the level of attitude has significant mean differences across gender, race, monthly income, school location, and source of knowledge from the Ministry of Education. The attitude scores highest with 93.8% compared to knowledge and practice for lifestyle characteristics of the school management (Table 3).

### Practice towards COVID-19 SOP compliance

The overall practice mean was 4.13 ± 0.422, and the scoring rate was 82.6%, suggesting that the respondents have a high level of practice towards COVID-19 SOPs. From a total of 740, 97.6% of respondents wear a face mask when going out (4.88 ± 0.377). However, this study findings surprisingly found that item P1 (I went on vacation during the outbreak) and item P2 (I went to any crowded place) have a low level of practice with a scoring rate of 32.4% (1.62 ± 0.961) and 36.7% (1.84 ± 0.946) respectively (Supplementary Table 5). The level of practice shows significant mean differences across gender, monthly income, school location, and source of knowledge from the Ministry of Education (Table 1). The practice also shows a significant difference in those who experience handling cases, managing programs related to infectious diseases, and being exposed to food poisoning (Table 2). Table 3 shows that practice score the lowest with 82.4% in lifestyle characteristics of the school management.

### Association between knowledge, attitude, socio-demographic, occupational, and lifestyle towards practice COVID-19 of SOP compliance

Based on the findings, there was a correlation between knowledge with practice (*p* < 0.001), attitude with practice (*p* < 0.001), and knowledge with attitude (*p* < 0.001). For the strength of correlation, the relationship between knowledge with practice was identified as weak correlation (r = 0.348), attitude with practice was very weak (r = 0.358), and knowledge with attitude was moderate (r = 0.619). Therefore, we accept the hypothesis that there was a significant positive relationship between knowledge, attitude, and practice toward COVID-19 SOP compliance by the school management (Supplementary Table 2).

Multiple linear regression analyses are provided in Table 4. The change in variance in practice explained by the independent variables is 23.4% (adjusted R-square). The table showed a significant positive influence of the independent variables toward practice (F = 13.231, *p* < 0.001). Seven independent variables had a significant influence on practice, which were knowledge (β = 0.332, SE = 0.083), attitude (β = 0.160, SE = 0.069), age (β = 0.168, SE = 0.045), physical activities (β = 0.108, SE = 0.038), Ministry of Health as a source of knowledge (β = 0.332, SE = 0.083), exposure to Tuberculosis (β = 0.123, SE = 0.083), and Measles (β = 0.110, SE = 0.083).

**Table 4:** Multiple linear regression (N = 740).

Variables	Unstandardized coefficient		Standardized coefficient	t	*p*-value	95% CI	
	B	SE	B			Lower	Upper
(Constant)	1.456	0.320		4.552	0.0001*	0.827	2.085
Knowledge	0.332	0.078	0.233	4.232	0.0001*	0.178	0.486
Attitude	0.260	0.064	0.216	4.042	0.0001*	0.133	0.386
Age (20–40 vs. 41–60)	− 0.080	0.036	− 0.098	− 2.225	0.027*	− 0.151	− 0.009
Source of Knowledge:							
a. Ministry of Health (Yes vs. No)	0.187	0.075	0.151	2.492	0.013*	0.040	0.335
Tuberculosis (Yes vs. No)	0.123	0.068	0.078	1.798	0.073*	− 0.011	0.257
Measles (Yes vs. No)	0.110	0.046	0.129	2.392	0.017*	0.020	0.201
Physical Activities (Yes vs No)	0.108	0.038	0.119	2.827	0.005*	− 0.182	− 0.033

R = 0.503, R Square = 0.253, Adjusted R Square = 0.234, F = 13.231 ANOVA *p*-value = 0.0001.

The regression model is:

$$\begin{aligned} {\text{Practice}}\left( {\text{y}} \right) & = {1}.{456} + 0.{332}\left( {{\text{knowledge}}} \right) + 0.{26}0\left( {{\text{attitude}}} \right){-}0.0{8}0\left( {{\text{age}}} \right) + 0.{187}\left( {{\text{source of knowledge}};{\text{ MOH}}} \right) \\ & \quad + 0.{123} + 0.{11}0 \, \left( {{\text{type of infectious diseases}};{\text{ Tuberculosis and Measles}}} \right) \\ & \quad + 0.{1}0{8 }\left( {\text{physical activities}} \right) \\ \end{aligned}$$

An increase in 1 standard deviation of knowledge will increase the practice by 0.332 standard deviations, while an increase in 1 standard deviation of attitude will increase the practice by 0.260 standard deviations. School management aged 41–60 have 0.080 practice more than school management aged 20–40. Physically active people have 0.108 practice more than those who are physically inactive.

## Discussion

The COVID-19 pandemic has had an impact on educational systems all around the world as a result of the closure of schools in the affected nations^19^. Nowadays, COVID-19 cases are still there, and many people continue to be infected thrice, even with complete vaccination, including boosters^20^. Therefore, SOP towards COVID-19 still needs to be maintained to prevent more cases from rising. During this pandemic, a few known, medically proven actions individuals can take to reduce their risk of catching the disease. Therefore, knowledge and attitude are expected to determine factors of practice toward COVID-19 SOP compliance.

The level of Knowledge, Attitude, and Practice (KAP) was measured based on three levels of the mean score; low (1.00–2.33), medium (2.34–3.67), and high (3.68–5.00). The overall knowledge mean score of the school management was 4.58 ± 0.304, slightly higher than the previous study by Chen et al. (2021), 4.46 ± 1.25. The slightly different might be because, after the COVID-19 arrival, people are pretty familiar with COVID-19. However, the knowledge score was slightly lower than the attitude score (4.72 ± 0.404) but slightly higher than the practice score (4.13 ± 0.422); contrast with a study among teachers in China showed that knowledge was slightly higher than practice and attitude was the lowest (Chen et al., 2021). The observed differences in knowledge, attitude, and practice scores between the current study and Chen et al. (2021) study highlight how public awareness and response to the changing COVID-19 pandemic situation are dynamic. The findings in this study suggest an increasing awareness and positive attitudes, potentially attributed to a more widespread understanding of COVID-19.

Our findings show that the school management has a high level of knowledge about COVID-19 (97.5%), COVID-19 transmission (97.5%), COVID-19 symptoms (95.1%), and COVID-19 standard operating procedures (85.5%), similar to the previous study by Abu Hasan et al.^8^. According to Mohd Hanafiah and Chang^21^, communication through various channels and public involvement during the COVID-19 pandemic has influenced Malaysian public knowledge, risk perception, and communication behaviours.

In this study, the school management often refers to the Ministry of Health and social media compared to other sources of information. According to Mohamad et al.^22^, most Malaysian refer to the Ministry of Health as the source of information since it is a local authority on public health^22^. Besides that, social media like Facebook also significantly influences the dissemination of information regarding COVID-19^23^. False information or rumours can heavily affect preventive behaviour toward COVID-19^24^. By interacting with school management via preferred means, such as social media, evidence-based guidelines can be effectively communicated, and a common understanding of preventive measures is fostered. Therefore, a reliable source of information during the pandemic is essential in maintaining compliance with COVID-19 standard operating procedures. In order to ensure that all school management is aware of accurate and updated information in the current rules, it is necessary to strengthen the methods of information dissemination.

Positive and significant relationships were discovered between knowledge and practice, attitude and practice, and knowledge and attitude among school management (*p* < 0.001). These ratings indicated that more people had excellent knowledge of and attitudes toward best practices for SOP compliance. In other studies^8,25–27^, a similar correlation between KAP was found, emphasizing the requirement to strengthen school management’s knowledge and experience in order to improve SOPs compliance. Those positive correlations highlight the significance of focused interventions meant to improve the knowledge and experience of the school management. The findings serve as the framework for comprehensive interventions that educate and encourage positive attitudes, which in the end create an environment where SOP compliance works effectively in the educational setting.

Knowledge, attitude, age, physical activities, source of knowledge, and exposure to infectious diseases were significant predictors of compliance with COVID-19 SOPs. Consistent with other research, people with higher COVID-19 knowledge significantly improved COVID-19 practice^12,28, 29^, Mat Din et al., (2020). The attitude toward COVID-19 was also discovered to be a strong predictor of excellent practice by Singh et al.^30^ and Zhong et al. ^31^. The study’s findings suggest that having good knowledge and attitude will improve practice for complying with COVID-19 SOPs^30,31^. As a result, since the SOP is entirely based on the risks in the area, risk communication needs to be adaptable and flexible to ensure its efficacy.

The level of SOP compliance was significantly greater among school management over 50. According to Chai et al.^26^, respondents between the ages of 18 and 40 received considerably lower scores for practices than Malaysians over 60 and those between the ages of 41 and 60^26^. An analysis of citizens from 12 Asian nations discovered a significant link between practice and the elderly population. Unlike other studies, they found no connection between practice and age^11,32, 33^. Therefore, although age appears to be a factor in SOP compliance, there are inconsistencies in the literature, highlighting the need for further research to understand the factors that influence compliance with SOPs across different age groups.

Interestingly, a high rate of SOP compliance was also significantly correlated with regular physical activity. There is not much research that links physical activity to COVID-19 preventative measures. A previous study observed that regular exercise is a practical method for preventing viral infection^34^. Exercise improves immune response and lowers the risk of severe COVID-19-related illness, hospitalisation, and even death^35,36^. Therefore, being physically inactive might impact COVID-19 SOP compliance in school settings,hence an intervention programme that encourages people to be physically active is required. These programmes could include physical fitness exercises, instructional initiatives, and campaigns that highlight the benefits of regular exercise in maintaining general health and improving adherence to COVID-19 SOPs.

Although the government has eased restrictions on interstate travel, school management should not relax their vigilance during the holiday seasons or anywhere else. Taking lessons from previous educational clusters in our country^6,37^, school administrators and teachers should take additional care when traveling and closely adhere to standard operating procedures.

Moreover, this study finding showed that the school management has high level of KAP. Other than that, those who have worked at schools located in Selangor and have higher incomes had a better mean score of knowledge, attitude, and practice. The school management also showed a positive and significant relationship between knowledge and practice, attitude and practice, and knowledge and attitude. Based on the significant predictors of practice, good knowledge of COVID-19, a positive attitude towards SOPs compliance, age above 50, experience in handling an infectious disease, exposure to infectious diseases, and being physically active are contributing factors to the high practice of the COVID-19 SOPs.

The significant predictors of practice that you've identified in this study can provide valuable insights for developing applications or interventions related to school management in the context of COVID-19. For instance, tailor training programs that emphasize the importance of good knowledge of COVID-19 and promote a positive attitude toward compliance with SOPs to specific age groups and roles within the school to maximize their impact. Other than that, create health and safety committees in the school with a representative combination of the students, staff, and teachers. These committees can supervise the execution and enhancement of SOPs and guarantee that they adequately address the requirements and apprehensions of the school community.

The strength of this study is one of the first to assess the KAP for COVID-19 SOP compliance in educational settings during the pandemic and might act as a standard for further studies. The majority of studies on KAP and COVID-19 concentrate on the patients as well as the pupils. The study will concentrate on COVID-19, particularly the SOP and the roles and duties of the school administration, including instructors. The survey was modified based on earlier research, following the most current MOE standards, and validated by subject matter experts. This study is a starting point for evaluating the effectiveness of the government’s prevention initiatives and identifying the locations and types of actions required to manage these diseases. Furthermore, regular physical activity and SOP compliance have been found to be significantly correlated in this study; this relationship has not been thoroughly explored in other studies.

It is essential to be aware of the limitations of this study. First, the results may not apply to school management at MOE-funded preschools or private schools because they were not included in this study. Second, the identified practice level might not entirely reflect their adherence to all SOPs in the guidelines because additional SOPs, such as SOPs for managing student residences, private institutions, and preschools, are not examined. Recollection bias could exist as a result of the self-reported nature of the questionnaire.

## Conclusion

The study’s findings show that school management has good knowledge of COVID-19 SOPs during the pandemic (91.6%), a positive attitude toward them (94.4%), and practices them (82.6%) effectively. In the interest of promoting high KAP toward the SOPs, health intervention programs targeting teachers and administrators in early adulthood, and early middle age, are essential, according to the significant predictors of practice identified in this study. Health authorities and policymakers can use the study’s findings to design intervention programs and improve existing SOPs for avoiding COVID-19 infections in schools in the future. It is also advised that MOE and MOH entirely use their official media to inform all active educational institutions about COVID-19 and SOPs and explain the risk effectively. Although our study indicates that the target population's Knowledge, Attitude, and Practice (KAP) levels are significantly high, the execution of health intervention programmes is still necessary for a number of reasons. Despite the fact the KAP scores are generally good, there might still be certain areas within these areas where focused improvements could lead to even better health outcomes. For instances, our study also demonstrates positive practices, there may be opportunity for targeted programmes to encourage even more reliable and effective health-related behaviours.

The identified variations in KAP, the impact of socioeconomic and demographic characteristics, the correlations between KAP variables, and the predictive capacity of particular factors serve as the foundation for the rationale behind the health intervention. These important factors can be taken into consideration while designing the intervention, ensuring a targeted and effective strategy for improving COVID-19 SOPs compliance within the school's management. Even though the COVID-19 pandemic has transitioned into an endemic phase, examining school management's adherence to COVID-19 SOPs is still important and relevant for ensuring a safe and healthy learning environment, responding to changing conditions, and being prepared for future health issues.

### Supplementary Information

Supplementary Tables.

## Data Availability

The datasets used and/or analysed during the current study available from the corresponding author on reasonable request.
