# Supplementary material for: Cracking the code: uncovering the factors that drive COVID-19 standard operating procedures compliance among school management in Malaysia
Source: Sci Rep. 2024 Jan 4;14:556. doi: 10.1038/s41598-023-49968-4 (PMC10766613; doi:10.1038/s41598-023-49968-4)
Supplement: Supplementary file 1 — Supplementary Tables. [file 41598_2023_49968_MOESM1_ESM.docx]

**Supplementary Table 1: The descriptive statistic of the KAP rating of the respondents (N=740)**

| **Variables** | **Mean±SD** | **Level** | **Scoring rate (%)** |
| --- | --- | --- | --- |
| **Overall Knowledge** | 4.58±0.304 | High | 91.6 |
| **Overall Attitude** | 4.72±0.404 | High | 94.4 |
| **Overall Practice** | 4.13±0.422 | High | 82.6 |
|  |  |  |  |

**Supplementary Table 2:** **Correlations between Knowledge, Attitude, and Practice (KAP) (N=740)**

|  | Knowledge |  | Attitude |  | Practice |  |
| --- | --- | --- | --- | --- | --- | --- |
|  | **r** | P-value | **r** | P-value | **r** | P-value |
|  |  |  |  |  |  |  |
| Knowledge | 1 | - | 0.619 | **0.0001*** | 0.348 | **0.0001*** |
|  |  |  |  |  |  |  |
| Attitude | 0.619 | **0.0001*** | 1 | - | 0.358 | **0.0001*** |
|  |  |  |  |  |  |  |
| Practice | 0.348 | **0.0001*** | 0.358 | **0.0001*** | 1 | - |
|  |  |  |  |  |  |  |
|  |  |  |  |  |  |  |

*Correlation is significant at p<0.05 (2-tailed).

**Supplementary Table 3:** Descriptive of Knowledge Towards COVID-19 SOPs Compliance (n=740)

| Items | Variables | Range | Mean±SD | Level | Scoring rate  (%) |
| --- | --- | --- | --- | --- | --- |
| Knowledge about COVID-19 | |  | **4.88±0.013** | **High** | **97.5** |
| K1 | COVID-19 is a viral infection | 1-5 | 4.88±0.380 | High | 97.6 |
| K2 | COVID-19 can cause a severe acute respiratory syndrome | 1-5 | 4.89±0.361 | High | 97.8 |
| K3 | The first case of human infection in Wuhan, China | 1-5 | 4.86±0.407 | High | 97.3 |
| Knowledge about COVID-19 Transmission | |  | **4.82±0.050** | **High** | **97.5** |
| K4 | COVID-19 can transmit through droplets during coughing or sneezing | 1-5 | 4.87±0.384 | High | 97.4 |
| K5 | COVID-19 virus can transmit via airborne | 1-5 | 4.74±0.663 | High | 94.8 |
| K6 | COVID-19 virus can be transmitted by touching surfaces and devices contaminated by the virus COVID-19 | 1-5 | 4.79±0.507 | High | 95.8 |
| K7 | COVID-19 can spread through close contact with infected persons | 1-5 | 4.87±0.383 | High | 97.4 |
| K8 | COVID-19 transmit via handshaking | 1-5 | 4.81±0.506 | High | 96.1 |
| K9 | Someone infected can transmit the virus even if they are not showing symptoms | 1-5 | 4.85±0.419 | High | 96.9 |
| K10 | Students, staff, etc., can wear face masks to prevent infection by the COVID-19 virus | 1-5 | 4.86±0.411 | High | 97.2 |
| Knowledge about COVID-19 Symptoms | |  | **4.76±0.152** | **High** | **95.1** |
| K11 | Fever, cough, and shortness of breath are COVID-19 symptoms | 1-5 | 4.84±0.445 | High | 96.8 |
| K12 | Symptoms of COVID-19 similar to the SARS virus | 1-5 | 4.53±0.756 | High | 90.5 |
| K13 | Patient with underlying chronic diseases is at higher risk of infection | 1-5 | 4.83±0.446 | High | 96.6 |
| K14 | People with age over 60 years old have a higher risk of developing severe diseases and death | 1-5 | 4.83±0.434 | High | 96.6 |
| Knowledge about COVID-19 SOP | |  | **4.28±1.038** | **High** | **85.5** |
| K15 | There is a COVID-19 SOP, particularly for an educational institution (school) | 1-5 | 4.90±0.329 | High | 98.1 |
| K16 | School management must conduct screening tests at the entrance | 1-5 | 4.56±0.834 | High | 91.3 |
| K17 | The school management must provide a Letter of Absence (LOA) to the student who is not allowed to school in relation to COVID-19 | 1-5 | 4.35±1.036 | High | 87.1 |
| K18 | School assembly is not allowed | 1-5 | 4.79±0.526 | High | 95.7 |
| K19 | Students or staff who have contact with someone infected should be immediately isolated. Close contact should remain in the isolation room for at least ten days or as directed by the PKD | 1-5 | 4.86±0.404 | High | 97.1 |
| K20 | Wearing a mask is compulsory around the school area | 1-5 | 4.90±0.356 | High | 98.0 |
| K21 | Cocurricular activities are not allowed in school | 1-5 | 4.69±0.683 | High | 93.8 |
| K22 | Student absenteeism during a pandemic is one of the discipline issues | 1-5 | 2.30±1.442 | Low | 46.1 |
| K23 | Students with symptoms are not allowed to attend school | 1-5 | 4.85±0.536 | High | 97.0 |
| K24 | Students are allowed to eat at the canteen during break | 1-5 | 2.11±1.381 | Low | 42.2 |
| K25 | School operation is based on phases in the National Recovery Plan (NRP) | 1-5 | 4.74±0.579 | High | 94.7 |
|  | **Overall Knowledge** |  | **4.58±0.304** | **High** | **91.6** |

**Supplementary Table 4:** Descriptive of Attitude towards COVID-19 SOPs Compliance (n=283)

| **Item** | **Item description** | **Range** | **Mean±SD** | **Level** | **Scoring Rate (%)** |
| --- | --- | --- | --- | --- | --- |
| AT1 | I believe good hygiene practices can prevent COVID-19 transmission. | 1-5 | 4.85±0.475 | High | 97.0 |
| AT2 | I am interested in knowing the COVID-19 preventive methods. | 1-5 | 4.73±0.544 | High | 94.5 |
| AT3 | I believe everyone is responsible for preventing the spread of the COVID-19. | 1-5 | 4.90±0.409 | High | 98.0 |
| AT4 | The Movement Control Order (MCO) is an effective effort by the government to curb the  spread of COVID-19. | 1-5 | 4.72±0.624 | High | 94.5 |
| AT5 | If I get infected with COVID-19, I will not do any high-risk practices that can get others infected. | 1-5 | 4.76±0.726 | High | 95.3 |
| AT6 | I agree that COVID-19 will be successfully controlled. | 1-5 | 4.63±0.639 | High | 92.6 |
| AT7 | I agree that compliance with SOPs can prevent COVID-19 transmission. | 1-5 | 4.82±0.483 | High | 96.4 |
| AT8 | I believe that vaccines can protect us against COVID-19 infection. | 1-5 | 4.55±0.744 | High | 90.9 |
| AT9 | The Ministry of Education has provided the educational institutions with adequate SOP and information on COVID-19. | 1-5 | 4.66±0.584 | High | 93.2 |
| AT10 | The SOPs provided are easy to understand and comply with by the school management. | 1-5 | 4.62±0.633 | High | 92.5 |
|  | **Overall Attitude** |  | **4.72±0.404** | **High** | **94.4** |

**Supplementary Table 5:** Descriptive of Practice towards COVID-19 SOPs Compliance (n=283)

| **Item No.** | **Item Description** | **Range** | **Mean±SD** | **Level** | **Scoring Rate (%)** |
| --- | --- | --- | --- | --- | --- |
| P1 | I went on vacation during the outbreak. | 1-5 | 1.62±0.961 | Low | 32.4 |
| P2 | I went to any crowded place | 1-5 | 1.84±0.946 | Low | 36.7 |
| P3 | I notify the district health office or specific parties if students or staff infected with COVID-19 | 1-5 | 3.20±1.67 | Medium | 63.9 |
| P4 | I wear a face mask when going out | 1-5 | 4.88±0.377 | High | 97.6 |
| P5 | I cover my mouth and nose with a tissue when coughing or sneezing | 1-5 | 4.66±0.604 | High | 93.3 |
| P6 | I wash my hands before and after each action | 1-5 | 4.67±0.554 | High | 93.4 |
| P7 | I stand 1-meter away from others (1-meter social distance) | 1-5 | 4.61±0.561 | High | 92.1 |
| P8 | I wash my hands with soap or hand sanitizer that contains at least 70% alcohol | 1-5 | 4.59±0.632 | High | 91.8 |
| P9 | I refer to the Standard Operating Procedure (SOP) issued by MOH, MKN, and MOE from time to time | 1-5 | 4.57±0.654 | High | 91.4 |
| P10 | I ensure all students, staff, etc. always wear face masks throughout the period of school | 1-5 | 4.66±0.550 | High | 93.3 |
| P11 | I supervise and ensure students or staff with temperatures above 37 °C or having any symptoms are not allowed to attend school | 1-5 | 4.46±0.863 | High | 89.3 |
| P12 | I gave a Letter of Absence (LOA) to students or staff whose infected with COVID-19 or under quarantine. | 1-5 | 3.06±1.663 | Medium | 61.1 |
| P13 | I ensure good ventilation in the classroom | 1-5 | 4.60±0.707 | High | 92.1 |
| P14 | I make sure that the table arrangement in the classroom is rotated | 1-5 | 4.62±0.702 | High | 92.5 |
| P15 | I supervise and ensure all the students eat in the classroom during break | 1-5 | 4.63±0.619 | High | 92.6 |
| P16 | I provide hand washing places with soap or hand sanitizer containing 70% alcohol | 1-5 | 4.53±0.786 | High | 90.6 |
| P17 | I ensure no mass gathering or school assembly within the school area | 1-5 | 4.59±0.774 | High | 91.8 |
| P18 | I ensure no curriculum activities are allowed in the school area | 1-5 | 4.53±0.839 | High | 90.6 |
|  | **Overall Practice** |  | **4.13±0.422** | **High** | **82.6** |
